# Occupation- and industry-specific cancer mortality among Japanese women from 1980 to 2015

**DOI:** 10.1186/s12889-022-14304-4

**Published:** 2022-11-01

**Authors:** Bibha Dhungel, Tomoe Murakami, Stuart Gilmour, Shunya Ikeda, Koji Wada

**Affiliations:** 1 Graduate School of Public Health, St. Luke’s International University, Tsukiji, Tokyo Japan; 2 Department of Health Policy, National Centre for Child Health and Development, Setagaya, Tokyo Japan; 3 Graduate School of Medicine, International University of Health and Welfare, 4-1-26 Akasaka, 107-8402 Minato City, Tokyo, Japan

**Keywords:** Mortality, Occupational mortality, Japan, Women, Cancer

## Abstract

**Background:**

The number of women in Japan who continue working after childbirth is on the rise. Over the past few years, Japan’s cancer mortality rate has increased. About 50% of all cancer deaths among Japanese women aged 25–64 are caused by lung, gastric, pancreatic and colorectal cancers. This study aims to examine the difference in mortality risk for key cancers among women and explore the effect of the economic crisis in the mid-1990s separately for occupational and industrial categories.

**Methods:**

Data from 1980 to 2015 were gathered from the Japanese Population Census and National Vital Statistics conducted in the same year. A Poisson regression analysis was used to estimate mortality risk and mortality trends for lung, gastric, pancreatic and colorectal cancer among Japanese working women aged 25–64 years.

**Results:**

Across most industrial and occupational groups, the trends in age-standardised cancer mortality rate for women have declined. Workers in management, security and transportation have a higher cancer mortality risk than sales workers. The risk of death from all four cancers is higher for workers in the mining and electricity industries than for wholesale and retail workers.

**Conclusion:**

To improve the health and well-being of employed Japanese women, it is crucial to monitor cancer mortality trends. Using these population-level quantitative risk estimates, industry- and occupation-specific prevention programmes can be developed to target women at higher cancer risk and enable the early detection and treatment of cancer.

## Background

Many Japanese women quit their jobs for childrearing. However, with the initiatives of the government focusing on flexible work environments and provision of paternal leave, the proportion of women continuing their employment is increasing. As Japan’s population rapidly ages the occupational and industrial structure, including the work environment, is changing. As one of the world’s largest industrial economies, Japan has many industries with a high risk of occupational exposure to environmental carcinogens, which is an important avoidable cause of cancer mortality [1]. Approximately 115,000 fatal and non-fatal accidents directly attributable to industry occur yearly in Japan, with one-third of the fatal accidents occurring in the construction sector [2]. According to the Global Burden of Disease Report 2019, occupational cancer causes 538,000 deaths annually [3]. Lung cancer, mesothelioma, leukaemia, ovarian and laryngeal cancer are some of the most common cancers related to occupational exposures among Japanese women [3]. Mortality disparities associated with environmental factors in different occupations and industries pose a significant occupational health challenge. Past studies have shown that occupation-specific mortality in Japan does not follow a clear socioeconomic gradient like its western counterparts [4] and white-collar workers such as professors and managers are known to have higher gastric, lung and cancer mortality rates than blue-collar workers [5–8].

The financial crisis in the late 1990s has led to an increase in this mortality inequality [5, 9]. The prevalence of cancer mortality in Japan has been rising in recent years. Smoking cigarettes, a family history of cancer, environmental factors such as dust, chemical exposure, and diet impose risk for lung cancer [10, 11] while age, sex, radiation, socio-economic status and alcohol consumption is associated with gastric cancer [12–14]. Modifiable risk factors like obesity, diabetes, smoking and alcohol consumption and exposure to pesticides, radiation and sedentary employment is associated with pancreatic cancer [15]. Alcohol consumption, smoking, and a variety of lifestyle and dietary factors increase the risk of colorectal cancer death, including lack of exercise, obesity, eating red or processed meat, and overconsumption of refined carbohydrates [16, 17]. There were 150,838 cancer deaths among Japanese women in 2015 [16], 2–5% of which were attributable to occupational exposure to carcinogens [18–21]. Lung, gastric, pancreatic and colorectal cancer were among the leading causes of cancer mortality among Japanese women aged 25–64 years and constitute approximately 50% of all cancer deaths. If detected at stage I, the 5-year relative survival rate of gastric and colorectal cancer in Japan is more than 95% followed by lung cancer at 81%, while that of pancreatic cancer is below 43% [16]. However, this rate decreases to 5%, 9%, 1.7% and 19% for lung, gastric, pancreatic and colorectal cancer, respectively if detected at stage IV. These figures make it even more important to detect these cancers at an early stage. Workers from specific occupations and industries have an elevated risk of cancer morbidity and mortality [22]. The Japanese government has set a priority to increase the cancer screening rates to greater than 50% [23, 24]. Nevertheless, the cancer screening rate in Japan remains the lowest among its OECD counterparts [25].

Around 70% of the female Japanese workforce is aged 25–64 years, and this proportion is increasing, with women also increasingly entering once male-dominated sectors in industry and transportation [26, 27]. These women are exposed to potentially hazardous carcinogens in the workplace. It is essential to analyse the risk among women due to the gender specific differences in workplace exposure, responses, carcinogenic potency and susceptibility. It is generally possible to characterize women’s risks based on the results of studies conducted on men, but this is not always the case [28], and past studies of occupational risk in Japan have often focused only on men. Recent studies have focused on occupation as a means of identifying women at high risk as a proxy measurement of health inequality [29]. However, most studies to date have explored leading causes of cancer by occupation and industry among Japanese men only [7]. A study by Okui explored trends in cancer mortality among manual and non-manual female occupational workers [8]. However, there is still insufficient evidence concerning the relative risk among Japanese women of developing lung, pancreatic, gastric and colorectal cancer in relation to occupational and industrial classification. This study will help in highlighting the association between socio-economic differences and cancer mortality. This study aims to examine the difference in mortality risk for key cancers among women and explore the effect of the mid-1990s economic crisis separately for occupational and industrial categories.

## Methods

A repeated cross-sectional study from 1980 to 2015 was conducted with five-yearly mortality data from the Japanese Population Census and the National Vital Statistics conducted in the same year. The government records the occupation and industry at the time of death, as reported by a family member of the deceased, and records the underlying cause of death from death certificates. We examined the four leading cancers – lung, gastric, pancreatic and colorectal cancer – in Japanese women aged between 25 and 64 years. For 1980, 1985 and 1990 we used the ninth revision of the International Classification of Disease, and the tenth revision for 1995, 2000, 2010 and 2015. Occupations were classified as: managers (administrative and managerial); professional; clerk; sales; service; security; agriculture; and manufacturing. The industry was classified as: agriculture and forestry; fishing; mining (mining and quarrying of stone and gravel); construction; manufacturing; electricity (electricity/gas/heat supply/water industry); transportation/communication (transport and postal activities/information and communications); wholesale/retail (wholesale and retail trade); finance (finance and insurance); real estate (real estate and goods rental and leasing business); service; and public services. Occupations and industries were classified according to the International Standard Occupational and Industrial Classification [30–33].

### Statistical analysis

Age-standardised rates were computed across occupations and industries using the five-year age-specific population of 1985 as a reference to analyse patterns in cause-specific mortality rates. Causes of deaths between 1980 and 1995 were coded based on the ninth revision of the International Classification of Diseases (ICD)-9, and ICD-10 codes for deaths after 1995. We used Poisson regression analysis to measure mortality risk and changes in mortality trends across occupations and industries separately by cancer type on or after 2000 compared to before 2000. Separate regression models estimated the incidence rate ratio (IRR) for lung, gastric, pancreatic and colorectal cancer separately by occupational or industrial category. Age was classified into four categories: 25–34, 35–44, 45–54 and 55–64 years. The estimated mortality rates were adjusted for age category, year, and a step variable in addition to the occupational or industrial classification. We used 1980 as the baseline (0), so that the intercept corresponds to 1980’s mortality rates. The step variable in the analysis indicated whether the mortality occurred from 1980 to 1995 or from 2000 to 2015, thus reflecting the change in mortality rates after the economic crisis in the late 1990s. A three-way interaction of year, step variable, and occupation or industry was included in the model separately for each cancer to reflect the changes in trends in mortality on or after 2000 compared to before 2000. We calculated the rate ratios as a linear combination of key variables with 95% confidence intervals for each occupational and industrial category to determine the changes in mortality trends and levels in 2000. All statistical analyses were conducted using Stata/MP 14.2 (Stata Corp, College Station, TX, USA).

## Results

A total of 24,347 cancer deaths among working Japanese women from 1980 to 2015 were analysed. Lung cancer accounted for 4543 deaths, gastric cancer 10,469 deaths, pancreatic cancer 3145 deaths, and colorectal cancer 6190 deaths among Japanese working women aged 25–64 years. Table 1 shows the age-standardised mortality rates per 100,000 population by occupation for lung, gastric, pancreatic, and colorectal cancer. The trends in age-standardised rates of lung, pancreatic, and colorectal cancer increased among managers from 1980 to 2015. Table 2 shows the trends in age-standardised mortality rates by industry for the key cancer causes. The absolute difference in lung and pancreatic cancer mortality among workers in the fishing and electricity industry increased between 1980 and 2015. Among workers in the mining industry, the age-standardised mortality rates decreased for lung, gastric, and pancreatic cancer, whereas it increased for colorectal cancer.

**Table 1:** Age-standardised mortality rates per 100,000 by occupation separately by cause of death from 1980 to 2015 among female Japanese workers aged 25–64 years

	1980	1985	1990	1995	2000	2005	2010	2015	Absolute difference^a^	1980	1985	1990	1995	2000	2005	2010	2015	Absolute difference^a^
**Occupation/cause**	**Lung cancer**									**Gastric cancer**								
Professional	3.8	4.1	5.9	4.7	5.6	3.3	3.6	1.9	-1.9	15.8	12.6	11.0	8.0	8.2	4.5	3.6	2.6	-13.2
Manager	9.8	11.7	19.7	15.1	17.3	16.9	14.6	20.3	10.5	26.8	39.5	27.8	24.6	28.9	30.1	25.3	10.3	-16.5
Clerk	5.1	3.6	2.8	2.9	2.1	1.2	1.3	0.9	-4.2	13.3	8.8	8.2	5.4	3.6	2.0	1.7	1.2	-12.1
Sales	4.4	4.7	4.7	4.0	3.6	2.5	2.3	1.6	-2.8	16.4	15.8	10.5	7.8	6.3	4.0	2.3	1.7	-14.7
Service	3.9	3.2	3.5	4.1	3.5	2.5	1.9	2.3	-1.6	13.4	10.1	9.2	7.6	6.0	3.2	2.7	2.5	-10.9
Security	47.6	38.6	50.9	22.5	21.9	17.8	8.6	18.1	-29.5	61.2	43.0	66.9	93.0	33.9	25.6	18.9	17.0	-44.2
Agriculture	4.5	4.4	4.6	5.7	4.2	3.4	3.9	2.0	-2.5	23.4	17.8	14.1	11.0	8.2	5.9	5.9	3.4	-20
Transportation	32.0	25.1	56.6	30.4	13.0	9.8	27.8	20.5	-11.5	92.8	63.4	76.4	17.4	24.8	20.3	16.7	15.9	-76.9
Manufacturing	1.8	2.2	2.0	1.4	1.4	1.1	1.1	1.1	-0.7	8.8	7.7	6.6	4.0	2.8	1.6	1.5	1.7	-7.1
**Occupation/cause**	**Pancreatic cancer**									**Colorectal cancer**								
Professional	4.7	2.4	3.2	2.2	3.0	2.6	3.0	2.1	-2.6	7.2	7.6	7.9	5.5	7.4	5.3	4.2	3.1	-4.1
Manager	2.1	3.7	7.7	6.2	6.0	14.2	15.3	8.5	6.4	10.8	14.8	22.5	17.4	22.2	15.2	25.3	15.5	4.7
Clerk	2.8	2.1	1.9	1.6	1.6	1.0	1.5	1.2	-1.6	4.5	5.2	4.2	3.1	2.4	1.8	1.8	1.3	-3.2
Sales	2.4	3.0	2.4	1.6	2.0	2.1	1.4	1.5	-0.9	6.2	5.6	7.2	5.5	3.9	3.6	2.7	2.4	-3.8
Service	2.4	2.2	2.4	3.0	2.3	1.8	1.6	1.6	-0.8	4.1	5.1	5.8	5.2	3.5	3.3	2.9	2.6	-1.5
Security	0.0	60.0	13.6	11.9	12.7	7.8	3.1	10.6	10.6	40.0	101.7	43.2	23.2	21.8	28.7	5.9	13.5	-26.5
Agriculture	3.6	3.3	3.0	3.1	3.3	3.7	4.7	1.4	-2.2	5.4	7.6	7.0	6.3	5.1	5.4	3.6	2.5	-2.9
Transportation	3.7	5.3	13.2	16.7	9.2	4.0	11.9	18.8	15.1	10.7	63.3	37.6	9.0	17.8	20.7	21.4	26.9	16.2
Manufacturing	1.4	1.4	1.4	1.3	1.0	0.8	0.9	0.9	-0.5	2.8	2.5	3.7	2.4	1.9	1.5	1.4	2.4	-0.4

^a^Absolute difference between 2015 and 1980

**Table 2:** Age-standardised mortality rates per 100,000 by industry separately by cause of death from 1980 to 2015 among female Japanese workers aged 25–64 years

	1980	1985	1990	1995	2000	2005	2010	2015	Absolute difference^a^	1980	1985	1990	1995	2000	2005	2010	2015	Absolute difference^a^
**Industry/cause**	**Lung cancer**									**Gastric cancer**								
Agriculture/Forestry	4.6	4.3	4.6	6.5	4.5	3.1	3.7	3.1	-1.5	23.4	18.0	15.5	12.1	9.6	6.3	6.7	4.2	-19.2
Fishing	4.5	4.8	9.3	13.0	5.9	20.2	11.8	23.2	18.7	41.6	28.8	21.3	29.3	19.6	13.5	21.9	26.5	-15.1
Mining	67.5	150.5	83.2	124.7	21.4	89.3	172.4	0.0	-67.5	278.3	191.1	277.6	190.4	124.8	271.7	134.3	55.0	-223.3
Construction	1.9	6.0	5.4	2.1	5.1	5.2	5.0	4.5	2.6	17.5	16.4	14.0	9.0	9.7	5.5	6.3	5.7	-11.8
Manufacturing	2.3	2.8	2.8	2.4	2.3	1.8	2.0	1.7	-0.6	9.9	8.9	7.6	5.3	4.5	2.8	2.5	2.5	-7.4
Electricity	14.8	32.5	57.2	36.7	40.5	14.6	34.1	30.2	15.4	87.3	124.0	135.9	65.4	58.3	46.2	17.4	15.8	-71.5
Transport/Communication	6.5	9.9	9.2	3.8	2.0	4.1	3.4	3.7	-2.8	33.5	24.3	15.1	7.4	5.6	5.3	3.3	3.0	-30.5
Wholesale/Retail	3.1	2.5	2.6	2.4	2.1	2.4	1.4	1.4	-1.7	10.0	8.5	6.1	4.5	3.5	3.3	2.0	1.6	-8.4
Finance	5.7	5.8	4.5	4.3	3.7	2.0	2.6	1.2	-4.5	17.1	18.0	11.4	6.6	3.8	3.4	2.5	1.8	-15.3
Real estate	2.5	6.4	7.3	3.2	4.5	2.1	3.1	1.8	-0.7	9.4	11.8	6.0	3.7	4.3	2.8	1.8	2.0	-7.4
Service	3.6	2.8	3.0	2.8	2.6	1.3	1.6	1.3	-2.3	12.3	9.0	7.5	5.2	4.2	1.9	1.9	1.5	-10.8
Public services	9.8	9.3	12.4	10.1	7.3	2.5	2.2	2.7	-7.1	35.9	28.3	24.7	17.6	11.8	3.8	3.5	2.1	-33.8
**Industry/cause**	**Pancreatic cancer**									**Colorectal cancer**								
Agriculture/Forestry	3.4	3.4	2.9	3.1	4.6	3.5	4.9	1.9	-1.5	5.4	7.6	7.4	7.1	5.8	5.3	3.4	2.5	-2.9
Fishing	5.5	4.5	2.8	6.5	3.7	6.1	3.1	13.3	7.8	9.8	8.2	10.4	8.0	15.1	8.5	9.4	8.6	-1.2
Mining	36.1	26.3	62.0	21.9	34.5	42.9	45.0	28.5	-7.6	135.6	148.0	122.2	88.5	64.4	183.4	154.2	212.4	76.8
Construction	3.7	3.6	3.6	1.7	2.0	3.3	3.7	2.9	-0.8	5.8	9.2	10.0	5.0	5.2	5.0	5.3	7.6	1.8
Manufacturing	1.4	1.7	1.7	1.7	1.5	1.3	2.0	1.5	0.1	3.6	3.0	4.4	3.4	3.0	3.1	3.0	3.0	-0.6
Electricity	0.0	5.6	28.4	10.0	29.0	54.4	9.3	27.4	27.4	43.4	35.3	127.4	26.9	54.2	49.3	27.4	20.9	-22.5
Transport/Communication	5.4	3.9	5.9	3.2	2.7	3.1	1.8	3.9	-1.5	7.9	15.0	8.8	3.6	4.2	7.0	4.7	4.6	-3.3
Wholesale/Retail	1.7	1.7	1.3	1.2	1.5	1.8	1.2	0.9	-0.8	3.7	3.4	3.7	3.1	2.0	3.1	1.6	1.7	-2
Finance	3.1	4.5	1.9	2.5	1.0	1.9	2.7	1.2	-1.9	6.9	8.7	6.0	3.8	2.9	2.7	2.4	1.9	-5
Real estate	0.9	2.6	2.3	2.0	1.8	1.9	3.0	2.7	1.8	2.6	3.6	6.7	3.6	3.5	3.8	2.0	1.8	-0.8
Service	2.5	2.1	2.3	2.0	1.3	0.8	1.4	1.2	-1.3	3.9	4.8	4.7	4.4	3.3	1.7	2.3	1.8	-2.1
Public services	8.7	3.9	4.3	5.0	6.5	3.2	3.0	2.2	-6.5	13.0	10.6	12.9	6.7	7.2	4.3	5.0	4.2	-8.8

^a^Absolute difference between 2015 and 1980

Table 3 shows the relative risk in mortality across the years by occupation and industry. Mortality risk among managers is higher than among sales workers for lung (IRR, 2.34; 95% confidence interval (CI), 1.67–3.28), gastric (IRR, 1.87; 95% CI, 1.52–2.29) and colorectal cancer (IRR, 2.04; 95% CI, 1.50–2.77) cancer. Similarly, mortality risk among security and transport workers is significantly higher than among sales workers for all four cancers.

**Table 3:** Mortality inequality across occupation and industry separately by cause of death among female Japanese workers aged 25–64 years

	Rate ratio (95% Confidence interval)				
**Occupational category**	**Lung cancer**	**Gastric cancer**	**Pancreatic cancer**	**Colorectal cancer**	
Professional	0.799 (0.602–1.062)	0.873 (0.759–1.005)	1.370 (0.992–1.891)	1.162 (0.938–1.439)	
Manager	2.340 (1.670–3.278)*	1.868 (1.522–2.293)*	1.638 (0.916–2.930)	2.043 (1.503–2.775)*	
Clerk	0.860 (0.692–1.069)	0.742 (0.662–0.832)	0.896 (0.669–1.201)	0.801 (0.664–0.967)	
Sales	Reference	Reference	Reference	Reference	
Service	0.801 (0.633–1.014)	0.728 (0.644–0.823)	0.818 (0.609–1.099)	0.742 (0.608–0.905)	
Security	25.615 (11.027–59.502)*	7.310 (3.735–14.306)*	69.252 (14.959–320.603)*	16.257 (8.001–33.031)*	
Agriculture	0.991 (0.818–1.199)	1.330 (1.208–1.464)*	1.313 (1.036–1.663)*	0.987 (0.838–1.164)	
Transport	4.351 (2.571–7.364)*	3.833 (3.016–4.871)*	6.650 (3.019–14.648)*	4.986 (3.307–7.518)*	
Manufacturing	0.451 (0.363–0.561)	0.539 (0.484–0.600)	0.561 (0.427–0.736)	0.488 (0.407–0.584)	
**Industrial category**					
Agriculture/Forestry	1.660 (1.366–2.017)*	2.338 (2.118–2.580)*	2.122 (1.664–2.707)*	1.699 (1.438–2.007)*	
Fishing	2.686 (1.391–5.186)*	3.679 (2.784–4.860)*	5.101 (2.499–10.411)*	3.029 (1.823–5.031)*	
Mining	35.614 (20.945–60.557)*	28.661 (21.160–38.820)*	41.887 (20.169–86.990)*	45.392 (28.777–71.601)*	
Construction	1.512 (1.058–2.162)*	1.886 (1.592–2.235)*	2.329 (1.560–3.476)*	2.321 (1.788–3.013)*	
Manufacturing	0.923 (0.740–1.150)	1.010 (0.902–1.130)*	0.936 (0.700–1.253)	0.924 (0.766–1.114)	
Electricity	12.916 (6.889–24.213)*	10.812 (8.023–14.572)*	33.403 (7.128–156.534)*	16.242 (10.093–26.140)*	
Transport/Communication	2.557 (1.710–3.822)*	3.048 (2.517–3.690)*	3.494 (2.157–5.658)*	3.305 (2.417–4.519)*	
Wholesale/Retail	Reference	Reference	Reference	Reference	
Finance	2.400 (1.660–3.470)*	2.007 (1.649–2.444)*	2.380 (1.470–3.852)*	2.285 (1.682–3.105)*	
Real estate	3.410 (1.841–6.316)*	1.363 (0.908–2.045)	2.235 (0.713–7.004)	1.795 (0.866–3.720)	
Service	1.123 (0.911–1.384)	1.205 (1.081–1.344)*	1.392 (1.071–1.810)*	1.171 (0.982–1.397)	
Public services	3.624 (2.635–4.983)*	3.838 (3.260–4.519)*	5.027 (3.411–7.407)*	3.917 (3.002–5.111)*	

*Significant at 5% level of alpha

Regarding industry, death from all four cancers for almost all industries except manufacturing is higher in comparison with the wholesale/retail industry. Workers in the mining and electricity industries have higher mortality rates than wholesale and retail workers.

Table 4 shows the trends in cause-specific mortality from 2000 to 2015 compared to 1980–1995, separately by industry and occupation. Trends in mortality by lung, gastric and colorectal cancer among professionals, managers, and sales and service workers decreased annually by over 3% on or after 2000 compared to before 2000. However, the trend in pancreatic cancer mortality among transport workers increased by 11% in or after 2000 compared to pre-2000.

**Table 4:** Change in trend of mortality rates by occupational and industrial category after 2000 for the leading causes of death

	Rate ratio (95% Confidence interval)			
Occupational category	Lung cancer	Gastric cancer	Pancreatic cancer	Colorectal cancer
Professional	0.926 (0.898–0.955)*	0.977 (0.956–0.998)*	1.021 (0.984–1.059)	0.970 (0.946–0.994)*
Manager	0.953 (0.914–0.993)*	0.964 (0.931–0.998)*	0.981 (0.922–1.044)	0.936 (0.900–0.973)*
Clerk	0.977 (0.951–1.004)	0.985 (0.966–1.005)	1.025 (0.992–1.058)	0.986 (0.963–1.010)
Sales	0.955 (0.930–0.981)*	0.963 (0.942–0.984)*	1.009 (0.975–1.044)	0.968 (0.946–0.991)*
Service	0.967 (0.942–0.993)*	0.973 (0.954–0.992)*	0.958 (0.929–0.988)*	0.958 (0.937–0.980)*
Security	1.041 (0.926–1.170)	0.982 (0.898–1.073)	1.071 (0.881–1.301)	1.033 (0.929–1.148)
Agriculture	0.987 (0.955–1.020)	0.995 (0.969–1.023)	1.001 (0.966–1.037)	0.963 (0.934–0.993)*
Transport	1.028 (0.953–1.108)	1.052 (0.990–1.117)	1.116 (1.004–1.242)*	1.032 (0.965–1.104)
Manufacturing	0.996 (0.967–1.025)	1.016 (0.995–1.037)	1.007 (0.974–1.040)	1.006 (0.983–1.030)
**Industrial category**				
Agriculture/Forestry	0.977 (0.946–1.009)	0.981 (0.956–1.007)	0.993 (0.960–1.027)	0.945 (0.917–0.974)*
Fishing	0.990 (0.902–1.086)	1.031 (0.961–1.107)	1.093 (0.957–1.247)	1.025 (0.927–1.134)
Mining	1.143 (0.972–1.343)	1.018 (0.934–1.109)	1.056 (0.893–1.249)	1.068 (0.978–1.165)
Construction	1.006 (0.962–1.052)	1.017 (0.986–1.049)	1.041 (0.986–1.099)	1.033 (0.997–1.071)
Manufacturing	0.981 (0.953–1.009)	0.992 (0.972–1.013)	1.000 (0.967–1.034)	0.989 (0.967–1.013)
Electricity	0.977 (0.900–1.061)	0.950 (0.886–1.019)	1.019 (0.856–1.213)	0.944 (0.878–1.015)
Transport/Communication	1.022 (0.965–1.083)	1.048 (1.004–1.093)*	1.059 (0.991–1.132)	1.033 (0.986–1.082)
Wholesale/Retail	0.979 (0.956–1.002)	0.996 (0.978–1.014)	1.001 (0.971–1.032)	0.992 (0.971–1.013)
Finance	0.988 (0.929–1.051)	1.032 (0.981–1.084)	0.998 (0.924–1.077)	1.026 (0.969–1.086)
Real estate	1.010 (0.927–1.101)	0.998 (0.922–1.080)	1.013 (0.887–1.157)	0.959 (0.872–1.054)
Service	0.966 (0.945–0.987)*	0.986 (0.970–1.003)	1.019 (0.993–1.046)	0.963 (0.945–0.981)*
Public services	0.912 (0.864–0.963)*	0.930 (0.889–0.972)*	0.958 (0.901–1.018)	0.997 (0.952–1.044)

*Significant at 5% level of alpha

Concerning industry, gastric cancer mortality increased by 4.8% among workers in the transportation and communication industries in or after 2000 compared to before 2000.

## Discussion

This study showed that trends in age-standardised cancer mortality rates in women are declining across most of the industrial and occupational categories in Japan. Managers and security and transport occupational workers have higher cancer mortality risk compared to sales workers. Similarly, workers from the mining and electricity industry have a higher mortality risk for all four cancers compared to wholesale and retail workers. For almost all occupational categories the mortality rates decreased faster after 2000 compared to before 2000. After 2000, pancreatic cancer mortality trends increased by 11% among transportation occupational workers compared to pre-2000. Similarly, compared to before 2000, gastric cancer mortality trends increased by 5% after 2000 among workers in the transportation and communication industry.

We found decreasing trends in age-standardised mortality rates for leading causes of cancer among Japanese women from 1980 to 2015 across occupations and industries. The overall result is similar to the findings from the Global Burden of Disease Report [34] and Monitoring of Cancer Incidence in Japan [35]. This could be related to the decreasing trends in smoking among women in Japan [36, 37]. Early detection of cancers such as gastric cancer and colon cancer through effective screening is a crucial factor affecting cancer mortality [38, 39]. A recent report showed that the participation of the Japanese working female population in cancer screening is increasing [40]. Furthermore, screening for and treatment of one of the major risk factors of gastric cancer [41], *Helicobacter pylori*, is now covered by health insurance in Japan [42, 43] leading to a decrease in *H. pylori* infection in the Japanese population [44]. These factors could possibly explain the decreasing mortality trends among Japanese women.

Studies have shown lower rates of stomach, lung [45] and gastric cancer [46] among workers from high socio-economic position. However, as similar to the findings for gastric cancer mortality by Yoshinaga et al., we found higher rates of all-four cancer mortality among workers from higher socio-economic class such as managers [6]. A higher risk of cancer mortality is also seen among security and transport occupational workers compared to sales workers. A stressful managerial job in Japan might lead to higher rates of cancer mortality [5]. The financial crisis of the late 1990s led to a decrease in the proportion of managers in the labour market from 3.8 to 2.6% between 1990 and 2010 [26]. With this reduction the work responsibilities of managers increased, leading to an increase in stress levels of managers [9]. Similarly, alcohol, smoking and dietary habits are known to be important risk factors for cancer [47]. Previous studies have shown that these risk factors are associated with lower socioeconomic status [48–50] which could possibly explain the higher cancer mortality among security and transport workers compared to sales workers. Additionally, as security and transport workers often work night shift [51], they could be at a higher risk of cancer mortality [52, 53]. Okui reported a high risk of cancer mortality in Japanese women with higher income [54]. The heavy work demand of managers may stop them from undertaking regular health checkups [55]. However, the increase in screening rate is prominent among manual workers while the participation rate remains low among non-manual workers [56, 57].

In Switzerland, a higher risk of lung cancer mortality is observed in the mining and construction industry for men and in the motor vehicle repair and driving industry for women [58]. The Occupational Disease Surveillance System showed that female workers in the construction, mining and electricity industries are at a higher risk of lung cancer and gastric cancer [46, 59, 60]. Similarly, the International Agency for Research on Cancer reported that lung and colorectal cancer are associated with coal dust [61]. Workers in these industries are known to have a higher overall cancer mortality risk because of the dusty working environment as well as continuous exposure to high levels of chemicals [60, 62]. The mining industry has a higher rate of dusty workplaces (50%) and carcinogenic substances (24%) than the sales industry [63]. Our findings suggest similar results, and showed that transport and security occupational workers and workers in the mining and electricity industries are at higher risk of cancer mortality than sales workers.

## Limitations

The Japanese dataset provided aggregated data by age category and does not provide individual-level data. Thus, we could not adjust for additional risk factors for cancer such as smoking and personal or family history of cancer. Many women may change their job after their cancer diagnosis, especially non-office workers. Thus, the occupation obtained from the certificate that provides the occupation or industry at the time of death may not reflect the nature of employment at the time of cancer diagnosis, which may affect the results. Additionally, the occupation and industry classification of the deceased was reported by the family of the deceased, which could have led to misclassification. The use of broad occupational and industrial categories in the current analysis might pose limitations in interpreting the result. Analysing the data with specific occupational and industrial categories would be helpful in better understanding the cancer mortality situation. The occupational and industrial classification has been revised over the years and may affect the mortality trend. Similarly, the transition of disease coding from ICD-9 to ICD-10 may also have affected the mortality trends. As women become an increasingly large proportion of the workforce and move into higher-risk manufacturing and industrial sectors, it is important to consider the economic cost of workplace-related mortality among women as well as men. This was not possible with the available data in the present study but should be considered in future research.

## Conclusion

Mortality among female workers in Japan differed considerably by occupation and industry from 1980 to 2015. The trends in cancer mortality, however, declined over the study period in most occupations and industries. It is vital to monitor cancer mortality trends for improving the health and well-being of employed Japanese women. The population-level quantitative risk estimates from this study can be used to implement industry- and occupation-specific prevention programs targeting women at higher risk of cancer for early detection and effective treatment.

## Data Availability

The data that support the findings of this study are available from Japanese Ministry of Health, Labour and Welfare but restrictions apply to the availability of these data, which were used under license for the current study, and so are not publicly available. Data are however available from the corresponding author upon reasonable request and with permission of Japanese Ministry of Health, Labour and Welfare.

## Abbreviations

CI: Confidence interval.
IRR: Incidence rate ratio.
